# Laser-based mid-IR photothermal spectroscopy of liquids: a new avenue for in-line sensing in process analytical technology

**DOI:** 10.1007/s00216-025-06000-0

**Published:** 2025-07-29

**Authors:** Dominik Kau-Wacht, Nelson G. C. Astrath, Gustavo V. B. Lukasievicz, Leopold Lindenbauer, Alicja Dabrowska, Karin Wieland, Bernhard Lendl

**Affiliations:** 1 Institute of Chemical Technologies and Analytics, TU Wien, Vienna, 1060 Austria; 2 Department of Physics, Universidade Estadual de Maringá, Maringá, 87020-900 PR Brazil; 3 Department of Physics, Universidade Tecnológica Federal Do Paraná, Medianeira, 85722-332 PR Brazil; 4 Competence Center CHASE GmbH, Vienna, 1030 Austria

**Keywords:** Infrared spectroscopy, Photothermal spectroscopy, Photothermal mirror, Photothermal beam deflection, Process analysis

## Abstract

Access to real-time chemical and physical information is of fundamental importance in modern producing industries, as it is needed for process monitoring and process control. It also enables process optimization, meeting regulatory requirements. This need motivates new developments in process analytical technologies. Optical in-line probes have emerged as powerful tools for non-invasive monitoring using a range of different spectroscopic techniques. In this regard, mid-infrared spectroscopy is of special interest as it can be used to retrieve both qualitative and quantitative information in a non-destructive and label-free manner. Recently, photothermal methods were also developed in the mid-infrared range, providing a high sensitivity and minimal sample preparation, making them ideal for detecting molecular and structural properties of gases, liquids, and in imaging applications. This study explores the application of reflection-based photothermal beam deflection (PTD) and photothermal mirror (PTM) spectroscopy in comparison with established fiber-optic-based attenuated total reflection spectroscopy (ATR) for real-time analysis of solutes in the mid-infrared range. Both techniques use the same ZnS window, incorporated in a flow cell for experimental simplicity and acting as the sensing interface. Furthermore, the presented PTM and PTD techniques also use the same excitation and probe lasers for ease of comparison. The results demonstrate the effectiveness of these techniques in detecting different concentrations of caffeine in chloroform with similar detection limits to previously presented approaches as well as a state-of-the-art commercial fiber-optic-based ATR process spectrometer. The investigated photothermal techniques hold promise for incorporation in a compact probe design void of any mid-IR fibers. This will pave the way for a new generation of rugged, sensitive, and long-term stable mid-IR in-line probes for use in demanding process analytical technology (PAT) applications.

**Graphical Abstract:**

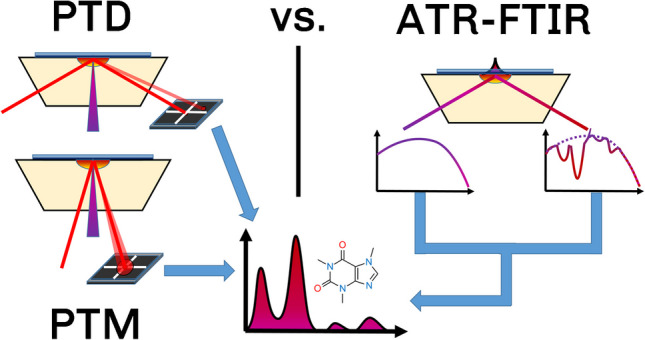

## Introduction

The real-time monitoring of chemical and physical properties throughout various operations in industrial and research environments is of fundamental importance and poses a real challenge in different areas. Optical in-line probes play an essential role in the PAT toolbox used in modern industries as they provide important information as required for process monitoring and control, thereby contributing to the improvement of process efficiency and maintenance of product quality and compliance with regulatory requirements [1–4].

The basic working principle for optical in-line probes depends on the effects of the interaction between light and matter. Several experimental methods based on absorption, scattering, and fluorescence can be used for precise chemical and physical characterization of substances without the requirement of sample extraction, thus allowing the detailed analysis of parameters like concentration, particle size distribution, molecular composition, and also of reaction kinetics in gases, liquids, and solids as well as in mixed phase systems [5].

Among the many optical techniques available, photothermal methods have gained significant attention due to their high sensitivity and ability to probe molecular and structural characteristics with minimal sample preparation. Photothermal techniques rely on light-induced heating, where absorbed optical energy leads to localized temperature changes, which modify the refractive index and cause thermal expansion and acoustic wave propagation. The thermal effects can be detected using optical methods such as interferometry, probe beam deflection, or infrared emission, providing valuable insights into material composition, molecular interactions, and thermophysical properties. These methods are particularly valuable in chemical and materials analysis, offering high spatial resolution and low detection limits. Early application of photothermal methods for gas and liquid sensing employing lasers in the visible and NIR spectral region had shown significantly improved sensitivities compared to their counterparts based on the more established absorbance spectroscopy. In liquid-phase analysis, photothermal lensing spectroscopy enables highly sensitive concentration measurements, surpassing traditional absorption techniques in detecting weakly absorbing species. However, it is interesting to note that these methods had found only limited adaptation in PAT applications despite their conceptual compatibility with turbid or highly scattering media where traditional optical methods like UV–Vis absorption spectroscopy may be less effective. In addition, these methods allow for the precise determination of the molecular composition, reaction kinetics, and thermal properties of liquids, solids, and nanomaterials [6–10].

Only recently, photothermal techniques have gained broad attention in analytical chemistry as modern mid-IR lasers such as broadly tunable external cavity quantum cascade lasers have been used as excitation sources. This has led to a revolution in mid-IR imaging technologies where localized absorption induced sample heating, causing also localized sample expansion, which can be probed via the cantilever of an atomic force microscope or via a visible probe beam, yielding spatial resolution of a few tens or hundreds of nanometers, respectively [11, 12]. The successful implementation of photothermal imaging systems beating the diffraction limit clearly demonstrates the maturity of these sensing modalities. With respect to gas and liquid sensing and using mid-IR lasers and employing photothermal sensing concepts and interferometer-based transducers, also interesting proof-of-concept studies were reported demonstrating high sensitivities. Employing interferometric cavity-assisted photothermal spectroscopy (ICAPS) which uses a Fabry–Perot interferometer as a transducer, quantification of SO_2_ in nitrogen down to the single-digit ppb concentration range has been achieved [13]. Water detection in organic solvents with detection sensitivities in the ppm range was shown using a Mach–Zehnder interferometer-based set-up on an optical bench [14].

Chemical analysis using optical in-line probes often involves identifying molecular structures, detecting contaminants, and quantifying component concentrations in real time. For instance, Raman spectroscopy provides molecular fingerprints based on vibrational energy shifts, making it a valuable tool for characterizing complex mixtures [15]. Similarly, NIR spectroscopy is widely used in pharmaceutical manufacturing for real-time monitoring of active pharmaceutical ingredient concentrations and blending uniformity [1]. In-line mid-IR sensing methods usually employ probes based on attenuated total reflection (ATR) Fourier transform infrared (FTIR) spectroscopy with chemically and mechanically stable internal reflection elements that come in contact with the sample. The limited sensitivity caused by the short interaction path length is typically increased by using multi-bounce elements. Deployment of claddings for analyte enrichment onto the surface of the ATR element may improve detection sensitivities; however, this concept has not yet been combined with in-line probes [16–21]. Recent work also shows that the sensitivity of in-line mid-IR and Raman probes towards particles (cells, polymers) in a suspension can be enhanced by ultrasound particle manipulation, which moves particles in and out of the probed volume in a controlled fashion [22, 23].

From a physical analysis perspective, optical in-line probes can assess turbidity, refractive index, and particle size distribution in colloidal suspensions or multiphase systems. Dynamic light scattering (DLS) and laser diffraction and scattering techniques are commonly applied for particle size measurements in chemical and food processing industries [24, 25]. Recently, photon density wave (PDW) spectroscopy has been introduced as a powerful in-line technique for particle sizing at high mass fractions [26]. In-line microscopy and focused beam reflectance measurements provide more detailed information on particle shape and morphology [27, 28]. Photothermal methods provide insights into material properties such as thermal conductivity, diffusivity, and structural heterogeneity. The photothermal mirror approach using a visible laser has been applied to quantitative analysis of the heat coupling between a solid and fluid, in addition to investigating the generation and detection of thermoelastic waves in metals and the effects of optical forces in dielectric liquids [29–31]. Furthermore, photothermal deflection techniques are applied in in-line particle size analysis and refractive index measurements, particularly in colloidal systems and emulsions [32], in addition to demonstrating the correlation between epidermal and blood-glucose levels in a type 1 diabetic patient [33].

Here, we propose a combination of all-optical non-contact reflection-based photothermal beam deflection (PTD) and photothermal mirror (PTM) spectroscopy as potential tools for real-time chemical and physical analysis of liquids in the mid-IR spectral range. The working principle of these photothermal methods is explained and compared with absorbance measurements. Both methods use the same sample cell configuration and excitation laser beam. Multiphysics simulations visualize heat transport and surface deformation of the flow cell window interfacing the sample. Different concentrations of caffeine in chloroform are investigated and the limits of detection are determined. The results are compared to a commercially available fiber-optic-based in-line ATR-FTIR spectrometer.

## Material and methods

**Fig. 1:**
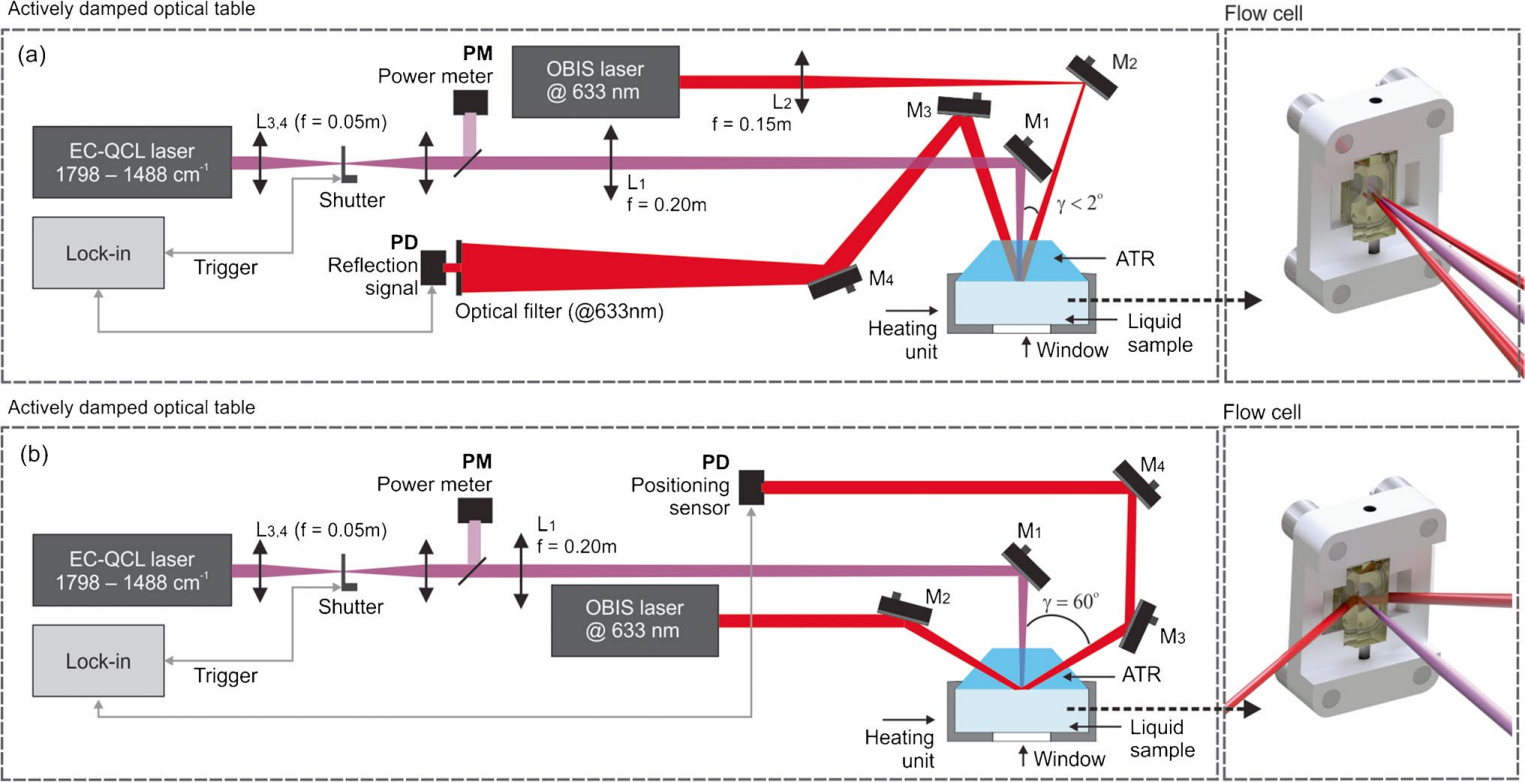
Schematic illustrations of the **a** photothermal mirror (PTM) and **b** photothermal deflection (PTD) geometry. ATR, attenuated total reflection crystal; EC-QCL, external cavity quantum cascade laser; f, focal length; L_i_, optical lens; M_i_, mirror; PD, photodetector; PM, power meter

Both experimental techniques used in this work being photothermal mirror (PTM) and photothermal beam deflection (PTD) are comparable since they use the same liquid flow cell configuration, excitation source, and all-optical pump-probe detection schemes. The sample cell consists of a CaF_2_ optical window (2 mm thick) and an attenuated total reflection (ATR) crystal (6 mm thick) separated by a liquid volume (2 mm thick). The ATR crystal is made of ZnS and has its wedged faces (30 arcmin) forming an angle of 60° with the base, such that it can be used either in the total internal reflection mode (PTD scheme) or in the normal reflection mode (PTM scheme), as shown in Fig. 1.

In both techniques, all-optical components are located on one side of the ATR window element, which also establishes contact with the sample. The basic difference between these techniques is the detection configuration of the probe beam. For the PTM technique, the probe beam is directed into the sample almost collinearly to the excitation beam. The probe beam, reflected off the interface between the sample and the ATR crystal, is propagated to the far-field detector plane. For the PTD method, the probe beam enters the ATR crystal by the faceted side and is reflected, propagating to the quadrature position-sensitive detector.

The excitation laser beam irradiates the sample perpendicular to the interface between the ATR crystal and the liquid sample, at the position where both the PTM and PTD probe beam spots are reflected. The ATR crystal and the back window are transparent to both the excitation and probe laser wavelengths, with the back window having no relevance neither for the PTM nor the PTD technique. Thus, only the liquid sample is responsible for the absorption of the excitation beam, inducing changes in the temperature and thermodynamic properties of the sample. As will be discussed in the next section in more detail, the accumulated heat is transferred to the ATR crystal and back window, resulting in thermoelastic deformations due to temperature changes. At the interface between the sample and the ATR crystal, the latter is deformed towards the former, creating a concave-like mirror to the probe beam, resulting in the focusing and deflection of the PTM and PTD probe beam, respectively. In addition, the temperature-induced change in the refractive index in the ATR crystal close to the sample contributes to the deflection in the PTD configuration due to the mirage effect and diverges the beam in PTM approach due to the photothermal lens.

A detailed description of the experimental apparatuses shown in Fig. 1 is given here: The excitation source was provided by a tunable external cavity quantum cascade laser (EC-QCL) (Hedgehog, DRS Daylight Solutions Inc., model 41,062-HHG-UT) emitting within the spectral range of 1798 cm^−1^–1488 cm^−1^ operated in continuous wave (CW) mode. The excitation beam was modulated at a frequency of 35 Hz and duty cycle of 50% using a mechanical shutter (Stanford Research Systems, Model SR475). The excitation beam was split at a ratio of 50:50 by means of a ZnSe beam splitter (Thorlabs, model BSW711). The reflected beam was measured by the infrared photometer PM (VIGO Photonics, model LabM-I-10.6) connected to a lock-in amplifier (Zurich Instruments, model MFLI 500 kHz) and used as a reference for the excitation power which varied across the excitation laser’s tuning range. The transmitted beam was focused on the sample position using a ZnSe lens (L_1_) with a focal length f = 0.20 m.

For the PTM configuration, a 35 mW continuous TEM_00_ solid state laser emitting at 633 nm (Coherent, model OBIS 633 nm LX 70mW), almost collinear to the excitation beam (γ < 2°), focused by lens L_2_ (f = 0.15 m), was used to probe the periodic deformation of the ATR/sample interface induced by the modulated excitation beam. The sample was placed close to the waist of the excitation beam at a position where the change in the radius of the excitation beam is minimal with respect to the change in the wavelength of the infrared excitation laser. The intensity of the probe beam center after reflection on the sample surface was maximized by adjusting mirror M_4_ and detected by a photodetector PD (Femto, Model OE-300-SI-10-FST, 200 MHz bandwidth). The lock-in amplifier demodulates the signal at both the photodetector and power meter using the frequency of the optical shutter as reference. A black-walled housing continuously flushed with dry air was used to prevent the ambient light from being detected by the photodetectors and reduce water vapor contribution as a source of noise in the spectral region under investigation. For each sample, an average of 5 spectra was computed. Each spectrum was recorded with the step and measure tuning mode of the EC-QCL controller with a step size of 1 cm^−1^ and stabilization time of 335 ms followed by an acquisition time of 30 ms per wavenumber step size. Each measurement takes 20 s. A time constant of 30 ms and filter order of 6 were used as parameters for the lock-in amplifier, with the trigger signal of the shutter used as reference. Data acquisition uses the demodulated signal amplitude from the two photodetectors to compute the probe signal normalized by the excitation power (PD/PM) as a function of the wavenumber.

For the PTD configuration, the same probe laser beam with a power of 10 mW enters the faceted interface of the ATR crystal (*γ* = 60°) where it undergoes a total reflection at the ZnS–liquid interface. Its periodic deflection induced by the modulated excitation beam is monitored by a quadrature position-sensitive photodetector (Thorlabs, Model PDP90A). All further acquisition settings and sensing configurations are the same as described for the PTM method.

Data acquisition and evaluation were performed using LabVIEW and Python, respectively. The Zurich Instruments LabOne API was used to control data acquisition, and laser control was based on the DRS Daylight Solutions Sidekick library. The Matplotlib package was used for plotting and the scipy.stats module for the linear regression. The Pandas and NumPy packages were used for parsing the data and performing data operations.

The samples used were prepared in concentrations of 0.1, 0.2, 0.5, 1.0, 1.5, 2.0, and 2.5 mg·mL^−1^ of caffeine anhydrous (99%, Sigma-Aldrich) in chloroform (CHCl_3_, anhydrous stabilized, VWR Chemicals). A stock solution of 25 mg·mL^−1^ was prepared in a volumetric flask. The samples were prepared by serial dilution by weighing the required amount of stock solution and the added chloroform to obtain reliable concentrations.

In order to compare the results of PTM and PTD to the state-of-the-art ATR-FTIR technique for in-line process monitoring, real-time in situ probing was performed using a commercially available ReactIR 15 FTIR process spectrometer (Mettler Toledo), which is equipped with a liquid N_2_-cooled MCT detector and connected to a multi-bounce diamond ATR probe via an AgX fiber of 1.5 m length. The dedicated ic IR 7.1 software (Metter Toledo, USA) was used for spectrometer control and data recording. Spectra were collected as a co-addition of 256 scans with a resolution of 4 cm^−1^ covering the spectral range from 3000 to 650 cm^−1^ taking 90 s per measurement. The collected spectra were exported as.csv files and evaluated similarly to the PTM and PTD.

The numerical simulations were performed with finite element analysis with realistic boundary conditions by using the software COMSOL Multiphysics 5.6.

## Results and discussion

### Fundamentals of the proposed photothermal techniques PTM and PTD

While the effects detected by the PTM and PTD techniques are different from the mid-IR ATR-FTIR technique, they also operate in a reflection geometry. Thus, the reflected beam retrieves information on the optical quality of the ATR/liquid interface as well as the time-dependent heat coupling effect within the liquid sample. These effects are enhanced at the surface due to the heat coupling and the associated thermoelastic expansion of the ATR crystal in contact with the liquid sample. In addition, scattering effects commonly encountered in transmission configurations are reduced.

The time-dependent heat coupling effect within the liquid sample and ATR crystal is dictated by the absorption of the liquid layer. In this case, the change in temperature ${T}_{i}$ in the liquid and in ATR caused by the laser absorption in the mid-IR range can be calculated by solving the heat diffusion equation [34]:

1 $$\begin{array}{c}\rho_ic_{pi}\frac{\partial T_i}{\partial t}-k_i\nabla^2T_i=Q_0\alpha_ie^{-2r^2/\omega_e^2}\cdot e^{-\alpha_i\left(z-z_i\right)}\end{array}$$

${{{\rho}}}_{{{i}}}$ is the mass density, ${{{c}}}_{{{p}}{{i}}}$ is the specific heat, $k_i$ is the thermal conductivity, $\alpha_i$ is the optical absorption coefficient at the excitation wavenumber, $\omega_e$ is the radius of the excitation beam in the liquid, and ${{t}}$ is the time. Since the problem is circularly symmetric, the temperature and displacement depend only on the normal z- and radial r-coordinates. The amplitude of the heat source is ${{{Q}}}_{0}=2{{{P}}}_{{{e}}}{{\phi}}/{{\pi}}{{{\omega}}}_{{{e}}}^{2}$. The optical absorption in the ATR can be neglected. $P_e$ is the excitation beam peak power, and ${{\phi}}$ accounts for the fraction of the absorbed energy converted to heat. When the absorbed energy is totally converted to heat then ${{\phi}}$ is 1.

The surface displacement in the ATR crystal, ${\text{u}}_{\text{i}}\left({{r}},{{z}},{{t}}\right)$, induced by the non-uniform temperature distribution, can be calculated using the thermoelastic equation [34].

2 $$\begin{array}{c}\left(1-2\nu_i\right)\nabla^2u_i+\nabla\left[\nabla\cdot u_i\right]=2\left(1+\nu_i\right)\alpha_{Ti}\nabla T_i\\+\;\frac{2\left(1+\nu_i\right)\left(1-2\nu_i\right)\rho_i}{Ei}\frac{\partial^2u_i}{\partial t^2}.\end{array}$$

is the Poisson’s ratio, is the linear thermal expansion coefficient, and is the Young’s modulus. The last term on the right side of (2) is known as inertia term. For continuous excitation and low modulation frequency, the inertia term can be neglected, and the displacement is governed by the temperature change. We applied finite element analysis (FEA) to solve (1) and (2). A complete FEA description can be found in [34]. The physical properties used for the simulations are given in [35].

**Fig. 2:**
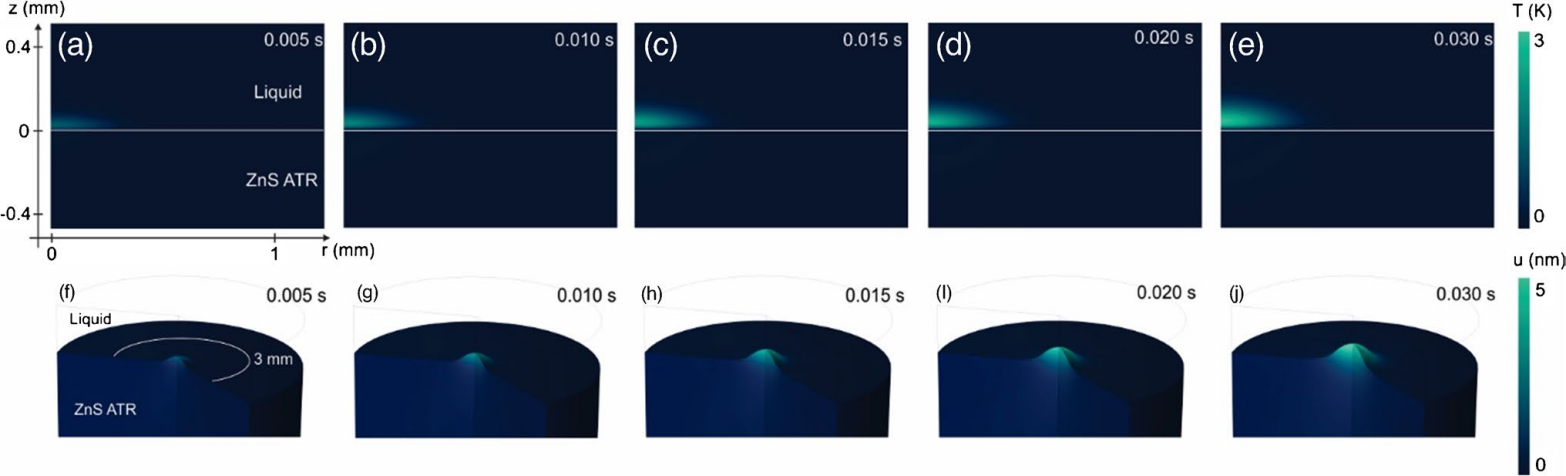
Time-dependent **a**–**e** temperature change and **f**–**j** surface displacement for different excitation time periods at the interface ZnS/liquid. ATR, attenuated total reflection crystal

Figure 2 shows the time-evolution of the temperature change and surface deformation in the interface between liquid sample and ATR crystal. Note that the laser-induced temperature change is mainly affecting the absorbing liquid, but by conduction, the ATR crystal also heats up. This heating changes the refractive index in the ATR crystal, altering the propagation of both probe beams from PTM and PTD. In addition, the temperature gradient in the ATR crystal results in a bulging of the ATR, which causes a surface deformation following the temperature change, as can be seen in Fig. 2f–j. As the probe beams for both methods are illuminated from the bottom, they sense a concave mirror being formed with time, which converges/deflects the probe beams at the ATR-liquid interface for the PTM and PTD, respectively.

### Experimental results for the PTM and PTD techniques and comparison with fiber-optic mid-IR ATR spectroscopy

**Fig. 3:**
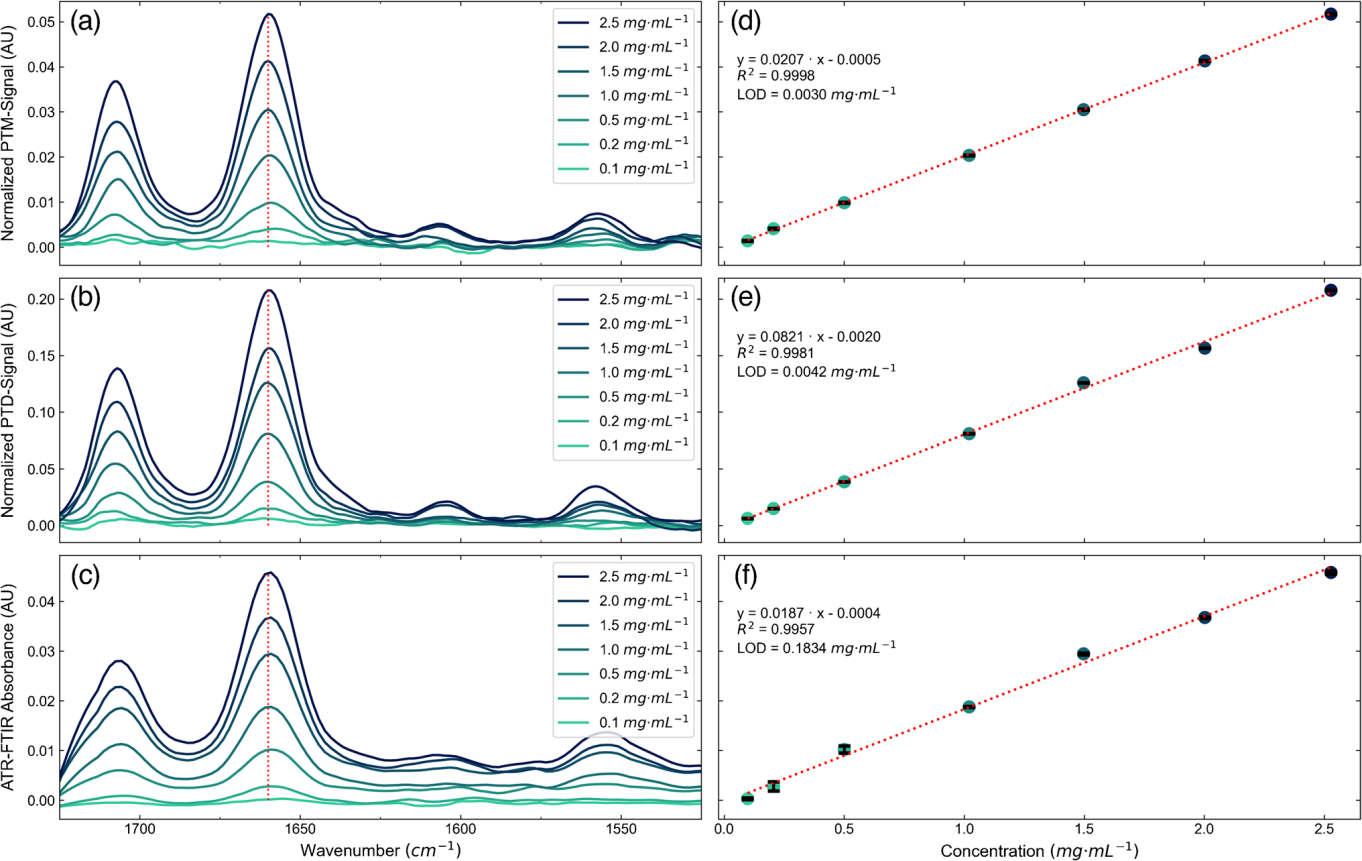
Normalized **a** photothermal mirror (PTM) and **b** photothermal deflection (PTD) and **c** attenuated total reflection Fourier transform infrared (ATR-FTIR) probe signals as a function of the wavenumber for different concentrations of caffeine in chloroform and the corresponding calibration curves for the **d** PTM and **e** PTD and **f** ATR-FTIR approach at 1660 cm^−1^. The spectra were smoothed using the Savitzky-Golay algorithm with second-order polynomial and 15 smoothing points

The prepared solutions of caffeine in chloroform were measured using the PTM and the PTD configuration as well as with the ATR-FTIR spectrometer. Figure 3a, b shows the PTM reflection and the PTD deflection spectra as a function of the excitation laser wavenumber. The PTM signal is obtained from the modulated variation of the center of the PTM probe laser beam at the far-field photodetector. For the PTD, the observed signal is the modulated deflection of the PTD probe laser beam at the position-sensitive photodetector. The obtained results agree well with the FTIR spectrum of caffeine in chloroform found in literature [36–38] and with the obtained results of the ATR-FTIR spectrometer seen in Fig. 3c. Two main absorption bands are displayed around 1700 cm^−1^ and 1660 cm^−1^, which correspond to in-phase C = O stretching and out-of-phase C = O stretching paired with C = C stretching, respectively [36]. Two bands of minor intensity around 1600 cm^−1^ and 1550 cm^−1^ may be attributed to C = C and C = N stretching vibrations and imidazole ring stretching vibrations in addition to stretching vibrations of CN, C = C, and CH bend, respectively [36, 37].

The obtained band heights at 1660 cm^−1^ were used to calculate the calibration curves shown next to the spectra (Fig. 3d–f). A linear regression was calculated for all approaches, and the measure of linear correlation was determined using the Pearson correlation coefficient. In addition, the limit of detection (LOD) and the limit of quantification (LOQ) were calculated for both detection methods following the procedure described in the literature [38], which uses the three times the standard deviation of a blank measurement as noise and the calculated slope of the calibration curve to obtain the LOD. It was already demonstrated that the Mach–Zehnder interferometry (MZI)-based photothermal spectrometer (PTS) shows a similar performance to a commercially available FTIR spectrometer with regard to noise level and LOD. In this work, we prove that both the PTM and PTD schemes have a similar performance with the important difference of an optical layout compatible with the requirements of an in-line probe, where all-optical elements are on the opposite side of the sample. A summary of the LODs in the literature as well as from the presented techniques is shown in Table 1.

**Table 1:** Comparison of the limit of detection (LOD) and limit of quantification (LOQ) for the photothermal mirror (PTM) and photothermal deflection (PTD) method as well as the attenuated total reflection Fourier transform infrared (ATR-FTIR) probe approach described in this work with the values for the FTIR and Mach–Zehnder interferometry-based photothermal spectrometer (MZI-PTS) reported in the literature [38]

Method	Noise (AU)	Slope (AU·mL·mg^−1^)	LOD (mg·mL^−1^)	LOQ (mg·mL^−1^)
PTM	2.1·10^–5^	0.0207	0.0030	0.0100
PTD	1.1·10^–4^	0.0821	0.0042	0.0140
ATR-FTIR probe	1.2·10^–3^	0.0187	0.1834	0.6115
FTIR	1.9·10^–4^	0.2606	0.0022	0.0073
MZI-based PTS	3.6·10^–4^	0.7165	0.0015	0.0051

LOD = 3·Noise/Slope, LOQ = 10·Noise/Slope

For the evaluation of the linear regression, the goodness-of-fit was evaluated according to DIN ISO 8466–1:2021, Annex A [39]. There, the residuals and the parameters of the calibration are used to determine the residual standard deviation ${s}_{y}$, the standard deviation of the method ${s}_{xo}$, and the coefficient of variation of the method ${V}_{xo}$. ${s}_{y}$ is calculated from the squared difference between the observed and calculated signals from the regression parameters. ${s}_{xo}$ is referencing ${s}_{y}$ to the slope of the calibration curve and ${V}_{xo}$ is relating ${s}_{xo}$ to the center of the working range $\overline{x }$.

The results obtained for the quality of the linear fit are summarized in Table 2 for the PTM and PTD method as well as the ATR-FTIR probe. While $s_{xo}$ measures the performance of a single analytical method, $V_{xo}$ can be used to compare different methods as it is a relative parameter dependent on the center of the working range. However, since both techniques are evaluated using the exact same solutions, only comparing $s_{xo}$ will have the same effect for the comparison. For the presented methods, the PTM approach shows the best performance with respect to the goodness-of-fit for the linear regression. However, both the PTM and PTD method show variation coefficients of less than 5%, which ensures a reliable quantification of caffeine in chloroform, while the ATR-FTIR based approach shows slightly poorer performance. This clearly highlights that the presented PTM and PTD methods perform similarly to the state-of-the-art ATR-FTIR process spectrometer. While it only takes approximately 20 s to collect a spectrum with the PTM and PTD method, co-adding 256 scans with the ATR-FTIR based approach takes 90 s. However, the latter has a broader spectral coverage.

**Table 2:** Comparison of goodness-of-fit parameters of the photothermal mirror (PTM) and photothermal deflection (PTM) method as well as the attenuated total reflection Fourier transform infrared (ATR-FTIR) probe approach. ${s}_{y}$, residual standard deviation; ${s}_{xo}$, standard deviation of the method; ${V}_{xo}$, coefficient of variation of the method

Method	${s}_{y}$(-)	${s}_{xo}$(mg·mL^−1^)	${V}_{xo}$(%)
PTM	3.4·10^–4^	0.0166	1.5
PTD	3.9·10^–4^	0.0475	4.2
ATR-FTIR probe	1.2·10^–3^	0.0635	5.7

$$S_y=\sqrt{\frac1{N-2}\cdot{\textstyle\sum_{i=1}^N}\left(y_i-{\widehat y}_i\right)^2}$$, $$S_{xo}=\frac{S_y}b$$, $$V_{xo}=\frac{S_{xo}}{\overline x}\cdot100$$

Typically, mid-IR-based methods used for sensing applications rely on the ATR measurements principle and the quantification of analytes using the Bouguer-Beer-Lambert law (3). There, the obtained absorbance $A(\overline{\nu })$ is a function of the wavenumber $\overline{\nu }$ and it is defined as the decadic logarithm of the ratio of the single beam background ${I}_{0}$($\overline{\nu }$) to sample signal $I(\overline{\nu })$ that is proportional to the molar decadic absorption coefficient $\varepsilon (\overline{\nu })$, concentration $c$ and the effective thickness $d\left(\overline{\nu },n\right)$, which in the case of the ATR technique is dependent on the wavenumber and the refractive index [40].

3 $$A(\overline\nu)=\log_{10}\left(\frac{I_0\left(\overline\nu\right)}{I\left(\overline\nu\right)}\right)=\varepsilon\left(\overline\nu\right)\cdot c\cdot d\left(\overline\nu,n\right)$$

When performing in-line mid-IR ATR absorption measurements, the background ${I}_{0}(\overline{\nu })$ and sample single beam spectra $I(\overline{\nu })$ are collected one after the other. In practice, the background can only be taken at the beginning of a reaction to be monitored. Therefore, the time lag between the reference spectrum and subsequent sample spectra increases with time, and so does the likelihood that the optical throughput of the whole sensing system changes due to external factors such as slight repositioning of the mid-IR fibers or temperature changes. This is problematic as such changes in throughput cannot be distinguished from changes in the sample single beam spectra caused by the ongoing reaction. Furthermore, the collected single beam spectra of the background and sample are required for calculation of the absorbance spectra, which are proportional to the analyte concentration and thus used for data analysis. The effect of limited sensor stability will be most problematic when industrial processes taking several hours need to be monitored. Furthermore, it is clear from (3) that an increase in power of the employed light source will not directly translate into increased absorbance values. Whereas some advantages may be realized when replacing the thermal source used in fiber-optic-based mid-IR ATR systems by a tunable laser, the increase in spectral power density will definitely not lead to a direct proportional increase in sensitivity as expressed in terms of the slope of a calibration curve. In contrast, for photothermal techniques the generated signal is proportional to the induced change in temperature $\Delta T$ that linearly scales with the concentration of the sample $c$, the absorption coefficient $\varepsilon$, the radiant power of the excitation source ${P}_{e}$ and with the inverse of the squared excitation beam radius ${\omega }_{e}$ (4). As depicted in Fig. 1, the radiant power of the employed source can be measured concurrently to recording the photothermal spectrum. Therefore, as opposed to mid-IR ATR spectroscopy, a continuous referencing of the recorded photothermal signals should be possible. Opposite to direct absorption spectroscopy, the photothermal signal can be improved by focusing of the excitation laser, thus paving the way for miniaturization towards on-chip sensors [6, 14, 40].

4 $$Signal\propto\Delta T\propto\frac{P_e\cdot\varepsilon\cdot c}{{\pi\cdot\omega}_e^2}$$

Furthermore, the absorbance linearly scales with the optical interaction path length, which is greatly limited when using ATR-based techniques as only the formed evanescent field penetrates the sample. Thus, while the sensitivity of the state-of-the-art ATR-FTIR spectrometer is limited, it can easily be improved for the photothermal techniques by employing more powerful light sources or by focusing the excitation beam. For the PTD approach, the sensitivity can be improved even further by increasing the distance between the detector and the sample, while it is fixed by the set geometry of the PTM method.

It is important to note that both PTM and PTD rely on the laser-induced excitation of the absorbing medium to generate the effects that are eventually probed in different ways by analyzing the reflection of the probe beam. This fact makes these techniques unique in the sense that the mechanisms associated with the signal generation are connected to the heating deposition in the sample and subsequent surface deformation of the ATR crystal. Conversely, these effects are essentially different from conventional transmittance/absorbance measurement methods, where the transmittance is recorded and compared to a reference measurement.

PTD and PTM techniques share many similarities, especially in the reflection-based ATR geometry using a modulated mid-IR EC-QCL source. However, they differ in what aspect of the thermal response they detect and in their sensitivity to certain physical parameters. PTD measures deflection of the probe beam due to temperature-induced refractive index gradients (mirage effect + thermal lens), while PTM is sensitive to change in curvature of the reflecting surface (interface) due to thermoelastic deformation + thermal lensing. The signal origin is primarily in the thermal boundary layer near the sample side of ATR for the PTD and dominated by surface deformation and lensing effect due to heat absorption in the sample. The signal interpretation requires careful modeling of heat-induced index gradients and probe path for the PTD and is more intuitive, as it relates to surface deformation and photothermal lensing for the PTM.

## Conclusion

We report on the use of reflection-based photothermal beam deflection and photothermal mirror spectroscopy for real-time chemical analysis in the mid-infrared spectral range. Both techniques share the advantages of operating in reflection mode while enhancing sensitivity due to the heat coupling effect at the ATR/liquid interface. The experimental results obtained from the optical setups confirm the capability of these methods for detecting different concentrations of caffeine in chloroform with detection limits similar to previous studies and a commercial instrument. Furthermore, separating the optical elements and the sample in a reflection-based configuration allows the integration of this method as an in-line sensor. Considering the overall dimension of modern room temperature operated external cavity quantum cascade lasers and also of laser arrays consisting of several single wavelength distributed feedback quantum cascade lasers on a single chip, a very compact probe design which does not require mid-IR fiber optics can be envisioned. These considerations further highlight the potential of mid-IR based photothermal techniques for industrial process monitoring, offering high sensitivity and minimal or no sample preparation. Future work may focus on the development of dedicated prototypes for in-line sensing and on expanding the application of these techniques to a broader range of chemical systems and industrial environments. For this, a specialized probe has to be designed that couples the probe visible laser beam at the specific angle for the PTM and PTD configuration as well as the pump quantum cascade laser beam into the ATR element.

## Data Availability

The data analyzed for the current report will be made available upon reasonable request.
